# Field Margin Plants Support Natural Enemies in Sub-Saharan Africa Smallholder Common Bean Farming Systems

**DOI:** 10.3390/plants11070898

**Published:** 2022-03-28

**Authors:** Baltazar J. Ndakidemi, Ernest R. Mbega, Patrick A. Ndakidemi, Steven R. Belmain, Sarah E. J. Arnold, Victoria C. Woolley, Philip C. Stevenson

**Affiliations:** 1 Department of Sustainable Agriculture, The Nelson Mandela African Institution of Science and Technology, School of Life Sciences and Bioengineering, Arusha P.O. Box 447, Tanzania; ernest.mbega@nm-aist.ac.tz (E.R.M.); patrick.ndakidemi@nm-aist.ac.tz (P.A.N.); s.e.j.arnold@greenwich.ac.uk (S.E.J.A.); 2 Agriculture, Health and Environment Department, Faculty of Engineering & Science, Natural Resources Institute, Medway Campus, University of Greenwich, Chatham Maritime, Kent ME4 4TB, UK; s.r.belmain@greenwich.ac.uk (S.R.B.); v.woolley291@gmail.com (V.C.W.); p.stevenson@kew.org (P.C.S.); 3 Royal Botanic Gardens, Kew, Richmond, Surrey TW9 3AE, UK

**Keywords:** aphid, conservation biocontrol, parasitoid, *Aphidius colemani*, floral resource plant, field margin

## Abstract

Flower-rich field margins provide habitats and food resources for natural enemies of pests (NEs), but their potential, particularly in the tropics and on smallholder farms, is poorly understood. We surveyed field margins for plant-NE interactions in bean fields. NEs most often interacted with *Bidens pilosa* (15.4% of all interactions) and *Euphorbia heterophylla* (11.3% of all interactions). In cage trials with an aphid-infested bean plant and a single flowering margin plant, the survival of *Aphidius colemani,* the most abundant parasitoid NE in bean fields, was greater in the presence of *Euphorbia heterophylla* than *Bidens pilosa*, *Tagetes minuta*, and *Hyptis suaveolens*. UV-fluorescent dye was applied to flowers of specific field margin plant species and NE sampled from within the bean crop and field margins using sweep-netting and pan-traps respectively. Captured insects were examined for the presence of the dye, indicative of a prior visit to the margin. Lady beetles and assassin bugs were most abundant in plots with *B. pilosa* margins; hoverflies with *T. minuta* and *Parthenium hysterophorus* margins; and lacewings with *T. minuta* and *B. pilosa* margins. Overall, NE benefitted from field margin plants, and those possessing extra floral nectaries had an added advantage. Field margin plants need careful selection to ensure benefits to different NE groups.

## 1. Introduction

The common bean (*Phaseolus vulgaris*) is important for food security for millions of people in sub-Saharan Africa (SSA). It is a major source of dietary protein, carbohydrates, and minerals [1], and contributes to soil fertility by fixing nitrogen with yield benefits for subsequent crops [2,3,4,5]. However, common bean production is constrained by several factors, including insect pests [6,7,8,9]. Synthetic pesticides are the primary technology used to manage bean pests, but this has adverse effects on human health, contributes to biodiversity loss, and leads to the resurgence of secondary pests [8,10]. More natural pest regulating approaches are required, such as conservation biological control, but this has not been adequately addressed in sub-Saharan Africa [11,12].

For example, natural enemies (NEs) that predate or parasitize insect pests can be a key component of sustainable pest management [13,14,15]. NEs benefit from non-crop plants in agricultural systems through the provision of shelter, nectar, and pollen for effective biological control [11,16,17]. It is possible to optimize the pest management contribution of NEs by managing field margin plants. However, to maximize this benefit, it is necessary to understand the specific advantages of each flowering plant to NE to improve natural pest regulation (NPR) and increase crop productivity. For instance, some flowers are tubular and lack nectaries, and these are not suitable for NEs [18,19,20,21,22].

Most studies focus on NE and the floral resource requirements in a specific context; either the benefit of the plants on NE in a controlled environment (e.g., cage), or whether the NE interact in the field with the margin plants and enhance NPR [11,12,16,17]. However, combining this information could help to better understand which plants will be most valuable in specific approaches supporting conservation biological control.

Field margin plants differ in their attractiveness to NEs [23], and few studies have been conducted on the contribution of these plants to supporting NEs of bean pests. Thus, we intended to test the following hypotheses: (1) field margin plants influence assemblage natural enemies on beans; (2) specific field margin plants influence differently the fecundity and survival of NEs on beans; and (3) NEs of bean pests use specific margin plants before migrating into the crop to provide NPR services.

## 2. Results

### 2.1. Interactions between Natural Enemies and Field Margin Plants

**Figure 1:**
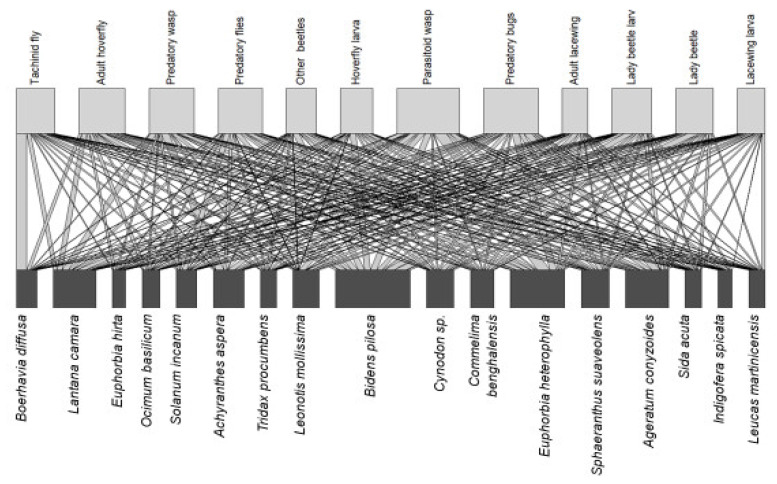
Interactions of natural enemies with field margin plants observed during transect walks in margins of bean fields over 12 months through visual observation, with *Bidens pilosa* having a high number of interactions with natural enemies compared to other field margin plants. The lower row shows plant species present, and the upper row shows the natural enemy guilds; the width of the linking bars indicates the frequency of the interactions observed.

This field trial surveyed the interactions of the field margin plant species with NE in bean fields. When observing NE-plant interactions on transect walks on bean fields, most insect groups investigated had interactions with most species of plant investigated. Overall, 5597 NE-plant interactions were observed, and the greatest number of interactions were recorded with the margin plant *Bidens pilosa* (861 interactions), followed by *Euphorbia heterophylla* (631). Parasitoid wasps were the NE group with the greatest number of observed interactions with field margin plants (724 interactions) (Figure 1).

### 2.2. Effects of Flowering Plant Resources on Parasitism and Survival of Aphidius colemani

**Figure 2:**
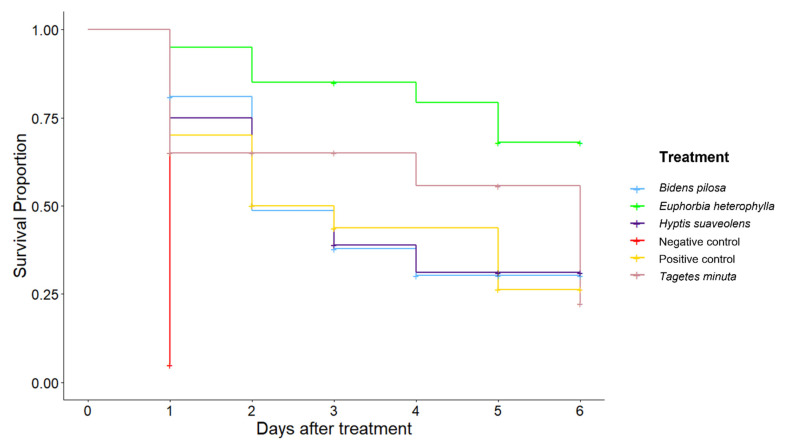
Survival of *Aphidiuscolemani* when provided different field margin plant species, sugar water (positive control), or only water (negative control). A ‘+’ represents a censored individual.

**Figure 3:**
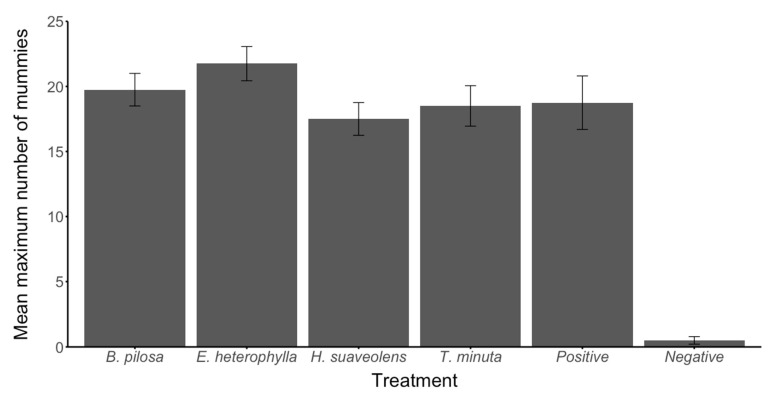
The mean number of *Aphis fabae* mummies produced per cage containing four females and two males of *Aphidius colemani*. Treatments: *B. pilosa*-*Bidens pilosa*; *E. heterophylla*-*Euphorbia heterophylla*; *H. suaveolens*-*Hyptis suaveolens*; *T. minuta*-*Tagetes minuta*; *Positive*-Positive control; *Negative*-Negative control.

In the controlled environment (cages), we investigated the potential of the key field margin plants to support the survival of the *Aphis fabae* parasitoid *Aphidius colemani* and optimize parasitism. When aphid-infested bean plants were caged with *A. colemani* parasitoids and a flowering margin companion plant, the survival of *A. colemani* was enhanced on all plant species compared to the negative control, demonstrating that nectar from all species tested supported NE survival. However, the effect differed significantly among the different flower treatments (*p* < 0.001; Figure 2). The plant that supported significantly improved survival of *A. colemani* compared to other plants, as well as the positive control, was *E. heterophylla*. There was no significant difference (F_4,16_ = 1.126, *p* = 0.381) between the number of mummies produced by *A. colemani* given access to any floral resource plants or the positive control (Figure 3).

### 2.3. Effect of Different Field Margin Plants in Supporting Natural Enemies in Bean Fields Fluorescent Dye

On a station trial, we cultivated bean plots surrounded by one of three field margin plant species, and treated the flowers with a fluorescent dye. We then captured insects in the crop (via sweep netting), and examined them for the presence of the dye, which identified the insects that had interacted with the field margin before moving into the crop, as well as pan-trapping within the margins. Lady beetles, hoverflies, assassin bugs, and lacewings differed in the number of fluorescent-labelled individuals captured the crop, according to the surrounding plant species (Table 1). Lady beetles, lacewings, and assassin bugs with fluorescent dye were particularly numerous in the *B. pilosa*-edged plots (and for lacewings, *Tagetes minuta*), indicating that these species regularly used *B. pilosa* before moving into the crop. Conversely, hoverflies were more numerous in plots surrounded by dye-marked *Parthenium hysterophorus* and *T. minuta*. Plots with *P. hysterophrous* margins were much less frequently used by lady beetles, lacewings, and assassin bugs (Table 1).

The number of natural enemies caught in pan traps in field margins also varied significantly depending on which field margin plant was present (Table 2), following similar patterns to the crop. Again, *B. pilosa* plots favored lady beetles, assassin bugs, lacewings, and parasitoid wasps within the margins, while *T. minuta* and *P. hysterophorus* favored hoverflies in the margins (Table 2).

The data for the field and laboratory trials are found in Supplementary Materials (Table S1).

## 3. Discussion

The composition of plants for a field margin that effectively supports natural enemies (NEs) may require different plant communities than for pollinators, though some plants may provide nectar and pollen to both natural enemies and pollinators [24]. The species that support NEs most effectively in East Africa are still poorly understood. Some common field margin species, such as *E. heterophylla* (Euphorbiaceae), *P. hyterophorus*, *B. pilosa*, *T. minuta* (Asteraceae), and *H. suaveolens* (Laminaceae), are invasive to SSA. However, their potential has been explored for pest control, pollination, and medicinal activities [11,16,25,26,27]. Our study aimed to determine which field margin plant species in SSA were beneficial to Nes in smallholder bean farms. The transect walk showed that Nes interact with multiple field margin plant species, although certain species had a higher number of interactions (*B. pilosa* and *E. heterophylla*). NEs depend on pollen and nectar from the plants, and plants provide alternative hosts in the absence of crops [28,29,30,31,32,33,34,35]. Similar results were found in a recent study by Arnold et al. [16] which showed that *B. pilosa* and *Euphorbia* sp. were preferred by natural enemies and pollinators in SSA. The use of *B. pilosa* and *E. heterophylla* by NEs could indicate that they provide valuable food resources or habitat. The observed interactions of NEs with *E. heterophylla* concur with the study by Patt [36] showing that this species provided nectar for lady beetles (*Coelophora inequalis*, *Cryptolaemus montrouzieri*, and *Harmonia axyridis*). Similarly, *B. pilosa* is effective in attracting populations of lady beetles (*Cycloneda sanguinea*) and hoverflies (*Pseudodoros* sp.) [37,38]. In addition, chemical cues from *B. pilosa* play a role in attracting natural enemies [39].

In station trials, we found that plots surrounded by *B. pilosa* margins were used frequently by lady beetles, parasitoids, and assassin bugs; *T. minuta* field margins were associated with catches of hoverflies, assassin bugs, lacewings, and parasitoids; and *P. hysterophorus* only with higher numbers of hoverflies and parasitoids. Furthermore, NEs caught inside the field crops with fluorescent dye indicated that the insects visited the flowers (possibly consuming nectar and/or pollen) before moving into the crop where they can provide pest control benefits. Relatively few long-legged flies and parasitoids were captured with the fluorescent dye, but this could be due to their small size rather than a lack of interaction with field margin species. Previous studies have shown the importance of *B. pilosa*, *T. minuta*, and *P. hysterophorus* in supporting NEs [27,37,38,39,40]. Floral resources from non-crop habitats are expected to support NPR by NEs [40,41,42,43,44,45,46]. Thus, selecting suitable plants for NEs is an important component of agricultural landscaping, as some plants will be better at supporting NEs. For instance, providing adult hoverflies with floral resources can enhance biological control by their larvae [47,48]. Moreover, pollen from some plants is superior to others in enhancing the performance of NEs [49], and this might explain why, in our data, one plant species, *E. heterophylla,* supported *A. colemani* better than the positive control.

Significantly more parasitoids were recorded in plots with field margin plants compared to the control (without field margin plants). In the transect walks, parasitoids were also the NE group with the most plant interactions. This could suggest that parasitoids are a NE group for which field margin plants are particularly important, providing carbohydrates, amino acids, and vitamins in nectar that enhance their pest controlling activities and optimize their metabolism [29,30,35]. This was demonstrated by using a cage trial experiment, which showed that all plants, i.e., *B. pilosa*, *H. suaveolens*, *T. minuta,* and *E. heterophylla,* resulted in improved *A. colemani* survival, and showed similar results to the positive control, which provided an in-cage carbohydrate food supply. Survival of parasitoids on *E. heterophylla* was greater than even the positive control and all other plants, suggesting that this species provided a greater nutritional benefit to *A. colemani*. Our results concur with similar studies, showing that access to flowers prolongs the lifespan and increases parasitism by *A. colemani*. For instance, studies on *A. colemani* and *Diadegma insulare* have shown improved performance compared with controls both in the field and when caged with flowering plant species such as *Fagopyrum esculentum*, *Conium maculatum*, *Photinia* × *fraseri*, *Brassica kaber*, *Barbarea vulgaris*, *Salvia apiana*, *Ligustrum japonicum*, *Lantana camara*, *Eriogonum fasciculatum*, *Daucus carota,* and *Thlaspi arvense* [50,51,52,53].

One of the reasons that *B. pilosa*, *H. suaveolens* and *E. heterophylla* supported *A. colemani* survival during the cage trial and that *B. pilosa* and *E. heterophylla* had high numbers of interactions with NEs in field trials could be the presence of extrafloral nectaries on these species [36,54,55,56]. Extrafloral nectaries are easily accessible, and the nectar composition differs from floral nectar and may be secreted differently [57]. Extrafloral nectar sugars are typically more concentrated than floral nectar, and normally it is present in larger volumes and secreted for a longer period [49,57]. Plants with extrafloral nectaries can be particularly important for NEs, as well as attractive to parasitoids [58], because these insects have mouthparts that are not suited to feeding on floral corollas; hence, they depend on plants with extrafloral or otherwise exposed flower nectaries [19,59,60]. Indeed, *E. heterophylla* produces extrafloral nectar right up to fruit maturation, possibly to provide food resources to attract natural enemies of seed and fruit pests [36].

Although *T. minuta* does not have extrafloral nectaries, it supported *A. colemani* survival in cage trials and plots, while *T. minuta* field margins had greater numbers of hoverflies, assassin bugs, lacewings, and parasitoids compared to control plots. This concurs with previous reports that *T. minuta* supports NEs. *T. minuta* increased the longevity of the egg parasitoid *Trichogramma minutum*, which enhanced the parasitism of the *Grapholita molesta* eggs [40]. Other species of *Tagetes*, including *T. erecta*, increased the longevity of *Cyrtorhinus lividipennis*, a NE of rice brown planthopper (*Nilaparvata lugens*) [61]. The extrafloral resources from the field margin plants could have additional benefits to NEs, and support biological control. Incorporating those field margins with extrafloral resources could bring positive effects for pest control in bean fields.

## 4. Materials and Methods

### 4.1. Interactions between Natural Enemies and Field Margin Plants

This field trial was carried out at Kwa Sadala Village in Hai District, Kilimanjaro region (3° 10′ 0″ S, 37° 10′ 0″ E). A total of eight sites with a high diversity margin and eight sites with a poor margin were visited. The high and poor diversity fields were determined by measuring the plant diversity in each farm before the selection of the sites. A transect walk along one margin, for the length of the field, was performed, and the visual observation of the NEs visits to the specific plant flowers was recorded. The sampling was conducted monthly during a year, and this coincided with specific bean crop development stages (1,5,9,14, etc., weeks after bean emergence).

### 4.2. Effect of Flowering Plant Resources on Parasitism and Survival of A. colemani

*Aphidius colemani* adults were obtained from *Aphis fabae* mummies collected from bean fields at Kwa Sadala in Hai District, Kilimanjaro region. *A. colemani* was selected for the cage trial because it has been reported as a primary parasitoid of *A. fabae* in SSA [12]. Moreover, this species is commercially produced for the biological control of many aphid species [62,63].

They were reared on potted bean plants infested with *A. fabae* in a wooden netted cage 30 × 30 × 60 cm. The plants were watered every three days. The *A. fabae* colonies were established from insects collected from farmers’ fields at Kwa Sadala village, the location of the field trials.

Bean seeds were grown in pots, then after five weeks, they were infested with 60 *A. fabae* (nymphs and apterous adults) [64]. The seeds from four field margin weeds (*Tagetes minuta*, *Hyptis suaveolens*, *Euphorbia heterophylla*, and *Bidens pilosa*) were germinated in pots before being planted out in fields. The experiment consisted of six treatments (*T. minuta*, *H. suaveolens*, *E. heterophylla*, *B. pilosa*, positive control, and negative control), and each treatment was replicated four times. Each cage contained one of these treatments with a potted bean plant infested with *A. fabae*. The positive control contained 10% sugar solution (glucose) as often as it was needed [65] and a potted bean plant infested with *A. fabae*, while the negative control had only a potted bean plant infested with *A. fabae*. Plants were watered every three days.

After leaving the aphids in the cage for 24 h to acclimatize, four female parasitoids and two male parasitoids were introduced to each cage. For the first seven days, the number of live parasitoids and mummies was counted daily to determine the survival of the first generation. Following this, the counting was performed three times a week. The number of parasitoids that emerged from mummies was recorded. The experiment was carried out for one entire lifecycle of parasitoids (approximately one month). The parasitoids and aphids were maintained under controlled conditions, with an average temperature of 25–27 °C, 66–68% R.H., and under natural lighting.

### 4.3. Effect of Different Field Margin Plants in Supporting Natural Enemies in Bean Fields

A field experiment was carried out at Kwa Sadala Village in Hai District, Kilimanjaro region, to monitor NE movement between field margins and crops. In total, three plant species introduced as above; *T. minuta*, *B. pilosa*, and *P. hysterophorus* were cultivated as the field margin. We initially selected *B. pilosa* and *E. heterophylla* due to the high number of interactions in the transect walk experiment. However, *E. heterophylla* failed to develop in the field, and thus other plants (*T. minuta*, *B. pilosa* and *P. hysterophorus*) were selected for subsequent field studies, as they occur frequently in SSA, have previously reported associations with beneficial insects [11,16,25,27], and are therefore straightforward for smallholders to acquire [26,66].

**Figure 4:**
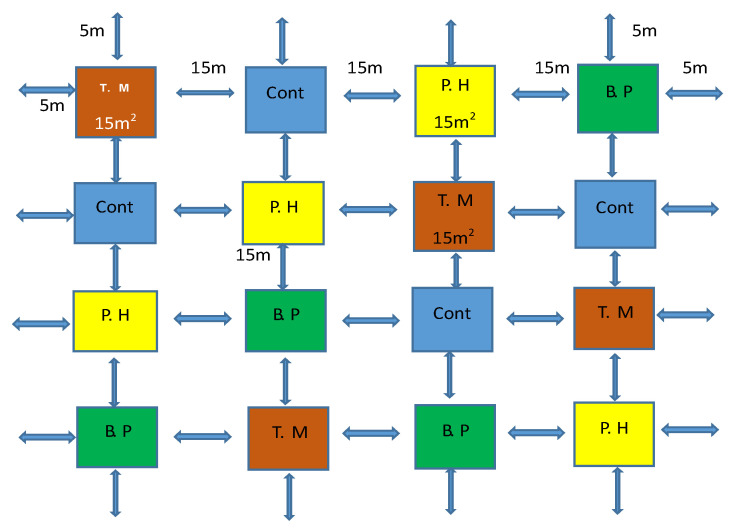
Field margin plants surrounding bean experimental plots for the fluorescent dye trial: T.M-*Tagetes minuta*; Cont-Control; P.H-*Parthenium hysterophorus*; B.P-*Biden pilosa*).

The experimental layout was composed of four treatment plots containing common bean (*Phaseolus vulgaris*) with four replications, 0.5 m field margin plants surrounded by three plots in each replication, each plot with the specific field margin plant, and the remaining plot was the control without a field margin. The plots measured 15 × 15 m, and the distance between plots was 15 m (Figure 4). During the flowering period of beans in the fifth week, powdered UV fluorescent dye (Baker Ross Ltd., Harlow, UK) was applied in the field margin plant flowers using a soft paintbrush. After 24 h, natural enemies were collected inside the field using sweep nets, then examined using a UV torch (365 nm; UVGear, Surrey, UK) to detect any fluorescent dye. This allowed the identification of insects that had visited the different field margin plants before being caught in the crop fields as an indication of the potential value of different species to different NEs. Pan traps were also used to collect NEs in the field margins [11]: two pan traps were placed in the field margin of each plot and natural enemies were sampled for three months (April, May, and June), coinciding with bean development stages. The collections were preserved in 70% ethanol for further identification.

### 4.4. Statistical Analysis

The interactions between NEs and different field margin plants were plotted using the ‘bipartite’ package [67] in R (RStudio Version 1.2.1335). Insects caught in field margins using pan traps were grouped into functional categories of NEs; catch distributions were checked for normality using a Shapiro–Wilk test. To assess the effect of field margin plants on the number of natural enemies caught, a histogram was plotted to assess the distribution of the data. Following this, GLM with Poisson distribution (RStudio Version 1.2.1335) was selected, as the data were not normally distributed. The fit of models was assessed with Chi-squared goodness of fit test, and all were found to follow this distribution. The month of sampling and field margin plant species were included in the GLM as covariates without interactions. Following this, pairwise comparisons were performed with the Holm multiple comparisons test in the ‘emmeans’ package [68].

The number of *A. colemani* surviving in cage trials was analyzed over six days using the Kaplan–Meier estimator of survival in R (RStudio Version 1.2.1335; [69]. For this analysis, surviving individuals were censored at the end of the experiment, and individuals were censored if it was not possible to monitor their survival for the duration of the experiment (e.g., for escaped individuals). Pairwise comparisons between treatments were then performed using a log-rank test with Benjamini–Hochberg correction. To assess the parasitism of *A. colemani*, the number of mummies in each cage was analyzed using ANOVA. Prior to the analysis, the negative control was removed due to lack of variance, and the normality of the remaining data was assessed using the Shapiro–Wilk test [50].

To analyze the number of insects labelled with the fluorescent dye and those captured in the field margin, GLM assuming Poisson distribution with a log link was used, followed by pairwise comparisons and a Holm multiple comparisons test. the normality was assessed using the Shapiro–Wilk test.

## 5. Conclusions

Flowering plants provide food and shelter for NEs and can promote natural pest regulation in crops. Our study highlights the potential of field margin plants in supporting populations of NEs in smallholder farms, and shows that conservation biological control could be used to promote NEs in these agro-ecosystems. Certain plant species appear to be preferred by different NE groups and provide different benefits. In transect walks, the highest number of Nes were observed interacting with *B. pilosa* and *E. heterophylla*. In addition, *B. pilosa*, *T. minuta*, and *P. hysterophorus* supported different groups of natural enemies when planted as a field margin. NE groups were shown to interact with flowers of these field margin plants, suggesting that they are supported by the provision of nectar and pollen. This is corroborated by cage trials where *B. pilosa*, *E. heterophylla*, *H. suaveolens*, and *T. minuta* enhanced the survival of *A. colemani*, most likely through the provision of nectar [27,37,39,40,70]. However, it is important to consider the wider implications of using these plants in conservation biological control, for example, *P. hysterophorus* is toxic [71,72], and other plants may be invasive to the area and present a challenge as weeds. Some field margin plant species might also provide food and shelter for specific pests, and therefore it is crucial to study the biology of the host plants and how they interact with pests.

## Figures and Tables

**Table 1:** Mean ± (SEM) numbers of natural enemies labelled with UV fluorescent powder within bean crops surrounded by different field margin plants. Different letters indicate significant differences between treatments within natural enemy groups. Significant differences were calculated using a GLM with Poisson distribution, followed by pairwise comparisons and a Holm multiple comparisons test.

Treatment	Mean Number of Natural Enemies (±SEM)					
	Lady Beetle	Hoverfly	Assassin Bug	Lacewing	Parasitoid Wasp	Long-Legged Fly
*Biden pilosa*	9.50 ± 2.02 a	2.50 ± 0.65 b	5.25 ± 0.95 a	6.50 ± 1.04 a	2.00 ± 0.82 a	1.50 ± 0.87 a
Control (no plant)	2.50 ± 1.04 b	5.50 ± 2.26 ab	0.75 ± 0.48 b	2.00 ± 0.41 b	1.75 ± 0.63 a	1.75 ± 0.48 a
*Parthenium hysterophorus*	4.25 ± 0.85 b	7.25 ± 0.48 a	2.25 ± 0.48 b	2.00 ± 0.82 b	2.25 ± 0.75 a	1.75 ± 0.48 a
*Tagetes minuta*	6.25 ± 1.03 ab	8.25 ± 0.85 a	0.75 ± 0.48 b	7.00 ± 1.47 a	3.25 ± 1.32 a	2.75 ± 0.48 a

Values followed by the same letters (a and b) within the column are not significantly different (*p* < 0.05).

**Table 2:** The mean ± (SEM) number of natural enemies caught in pan traps in field plots with different field margin plants. Different letters indicate significant differences between treatments within natural enemy groups. Significant differences were calculated using a GLM with Poisson distribution, followed by pairwise comparisons and a Holm multiple comparisons test.

Treatment	Mean Number of Natural Enemies (±SEM)					
	Lady Beetle	Hoverfly	Assassin Bug	Lacewing	Parasitoid Wasp	Long-Legged Fly
Control (no plant)	1.83 (± 0.63) a	1.92 (± 0.54) a	1.25 (± 0.70) a	1.50 (± 0.86) a	1.75 (± 0.49) a	1.75 (± 0.63) a
*Bidens pilosa*	5.92 (± 1.05) b	2.67 (± 0.77) ab	3.75 (± 0.35) b	3.50 (± 0.42) b	5.31 (± 1.53) b	1.67 (± 0.45) a
*Parthenium hysterophorus*	2.17 (± 1.95) a	4.50 (± 0.82) b	2.42 (± 0.78) ab	2.42 (± 0.86) ab	3.23 (± 0.93) b	1.08 (± 0.34) a
*Tagetes minuta*	3.33 (± 0.88) a	4.58 (± 0.83) b	3.42 (± 1.23) b	3.58 (± 0.93) b	4.58 (± 1.32) b	0.75 (± 0.22) a

Values followed by the same letters (a and b) within the column are not significantly different (*p* < 0.05).

## Data Availability

Data is contained in Supplementary Materials.

## Supplementary Materials

The following supporting information can be downloaded at: https://www.mdpi.com/article/10.3390/plants11070898/s1, Table S1: Experimental data.
